# Functional integrity of the SEL1L–HRD1 complex is critical for endoplasmic reticulum–associated degradation and organismal viability

**DOI:** 10.1073/pnas.2517927123

**Published:** 2026-02-05

**Authors:** Xiawei Zhang, Liangguang Leo Lin, Linxiu Pan, Xiaoqiong Wei, Huilun Helen Wang, Zexin Jason Li, Ling Qi

**Affiliations:** ^a^Department of Molecular Physiology and Biological Physics, University of Virginia School of Medicine, Charlottesville, VA 22903

**Keywords:** ERAD, SEL1L variants, SEL1L–HRD1 interaction, ER quality control, neonatal lethality

## Abstract

The SEL1L–HRD1 complex is the most conserved branch of endoplasmic reticulum–associated degradation (ERAD), yet whether SEL1L is strictly required for HRD1 function in mammals has remained unclear. Here, we show, using complementary in vivo and in vitro approaches, that direct SEL1L–HRD1 binding is essential for ERAD activity and neonatal survival. Three knock-in mouse models targeting this interface reveal a clear genotype–phenotype relationship: the L709P variant, which abolishes SEL1L–HRD1 association, causes complete neonatal lethality; the partially disruptive S658P variant results in partial lethality; and the P699T mutation preserves the interaction and yields normal viability. Mechanistically, our data show that the SEL1L–HRD1 interface is essential for ERAD complex formation and activity, enabling both substrate handoff and E2 enzyme recruitment, and that the L709P mutation effectively uncouples these core steps of the mammalian ERAD pathway. These findings establish SEL1L–HRD1 coupling as a core requirement for mammalian ERAD function and early postnatal viability.

## Significance

Endoplasmic reticulum–associated degradation (ERAD) is fundamental to cellular and organismal survival, yet the molecular determinants that enable a functional mammalian ERAD complex have remained unresolved. By combining mouse genetics with mechanistic studies, we show that direct binding between SEL1L and the E3 ligase HRD1 is indispensable for ERAD function and neonatal viability. Mutations that weaken or abolish this interaction produce graded defects in misfolded protein clearance and organismal survival. These findings define SEL1L–HRD1 association as an essential element of mammalian ERAD and illuminate how disruption of this interface contributes to early lethality and disease.

In eukaryotic cells, approximately one-third of nascent proteins enter the endoplasmic reticulum (ER), where they fold and mature into functional conformations (1, 2). Misfolded proteins—arising from genetic mutations or folding inefficiencies—are eliminated by ER-associated degradation (ERAD), a conserved quality control process that targets them for cytosolic proteasomal degradation (3–9). The most conserved ERAD branch involves the Hrd3p–Hrd1p complex in yeast and its mammalian counterpart, the SEL1L–HRD1 complex (10–14). Genetic ablation of *Sel1L* or *Hrd1* in mice, either germline or induced in adults, leads to embryonic or premature lethality, respectively (15–18). Cell-type-specific deletions have revealed broad roles in diverse physiological processes, including nutrient and energy metabolism, and immune regulation (19–35). Consistent with these fundamental functions, biallelic variants in *SEL1L* and *HRD1/SYVN1* were recently linked to human neurodevelopment disorders known as ERAD-associated neurodevelopment disorders with onset in infancy (ENDI) syndrome (36, 37).

Both yeast Hrd3 and mammalian SEL1L function as scaffolds that stabilize HRD1 and organize its interactions with key ERAD cofactors, including the lectin OS9, E2 enzyme UBE2J1, and DERL (18, 38–44). Intriguingly, Hrd1p overexpression in yeast can bypass the requirement for Hrd3p (45–47), suggesting that the E3 ligase can, under certain conditions, operate independently of its canonical adaptor. In mammals, proteomic studies have likewise identified SEL1L-independent HRD1 assemblies, including complexes containing FAM8A1 (48, 49). Furthermore, recent work indicates that HRD1 may support TLR3 trafficking to endolysosomes in a SEL1L- and ERAD-independent manner (50), and SEL1L has been implicated in HRD1-independent functions such as cell-mediated collagen clearance (51). Together, these observations raise a fundamental question: To what extent do SEL1L and HRD1 function as an obligate pair in mammals, and can either component execute independent biological roles? Despite extensive biochemical characterization of ERAD machinery, the physiological necessity of the SEL1L–HRD1 interaction has remained unresolved (52).

We previously reported that the SEL1L S658P variant, originally identified in Finnish Hounds with cerebellar ataxia (53), lies near the SEL1L–HRD1 interface, partially weakens their association, and causes a similar neurodegenerative phenotype in mice (44). Homozygous S658P knock-in (KI) mice survive to weaning at sub-Mendelian ratios (~11%) and develop early-onset ataxia in adulthood (44). Here, we generated two additional KI mice carrying human SEL1L missense variants, L709P (NM_005065, c.2126T>C; p.Leu709Pro) and P699T (c.2095C>A; p.Pro699Thr), both mapping directly to the SEL1L–HRD1 interface. Together, the P699T, S658P, and L709P KI models reveal a coherent structure–function–physiology relationship: The extent of SEL1L–HRD1 disruption corresponds directly to ERAD impairment and stratifies organismal survival.

## Results

### Generation of Two KI Mice Carrying Human SEL1L Variants.

**Fig. 1.**
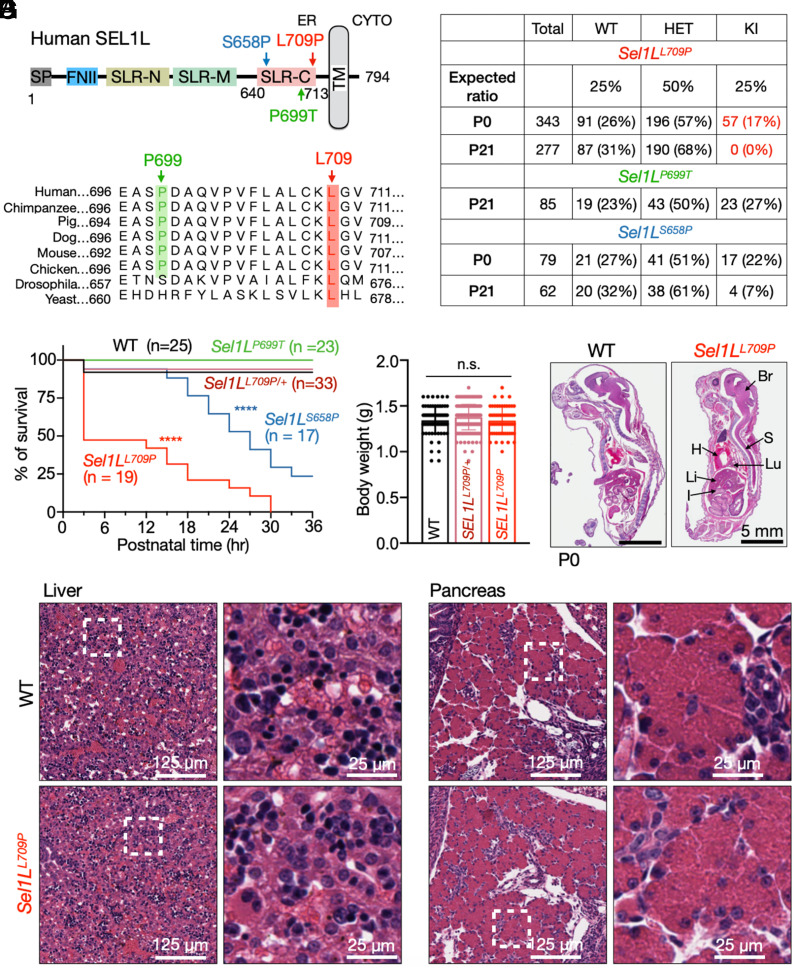
Homozygous *Sel1L^L709P^* KI mice exhibit neonatal lethality. (*A*) Schematic diagram of the human SEL1L protein domain structure, highlighting the position of three variants at SLR-C. SP, signal peptide; FNII, fibronectin type II domain; SLR-N/M/C, N-, middle-, and C-terminal Sel1-like repeats; TM, transmembrane domain. (*B*) ClustalW sequence alignment demonstrating evolutionary conservation of residues L709 (red) and P699 (green). (*C*) Number and percent of mice of each genotype at postnatal (P) days 0 and 21. (*D*) Kaplan–Meier survival curves for neonates over the first 30 h after birth. n, mouse numbers. ****P < 0.0001 (comparing L709 or S658P KI to WT or P699T KI mice) by Log-rank (Mantel–Cox) test. (*E*) Body weights of WT and KI neonates at P0. Data, mean ± SEM; n.s., not significant by one-way ANOVA with Dunnett’s multiple comparisons test. (*F* and *G*) Hematoxylin and eosin (H&E) staining of WT and *Sel1L^L709P^* KI P0 pups, with livers and pancreas shown in *G*. Br, brain; S, spinal cord; H, heart; Lu, lung; Li, liver; I, intestine. n = 3 mice per group.

We identified two heterozygous SEL1L missense variants, P699T and L709P, through a search of clinical exome and genome sequencing data from the Baylor Genetics Laboratory. Although these variants were considered unlikely to be disease-causing—because they were heterozygous and occurred in patients without phenotypes consistent with ENDI syndrome (36, 37, 52)—we prioritized these variants because both lie within the highly conserved C-terminal Sel1-like repeat (SLR-C) domain of SEL1L (Fig. 1*A*)—the region that engages HRD1—and each affects highly conserved residues, especially L709P (Fig. 1*B*). To assess their functional significance in vivo, we generated knock-in mice carrying the orthologous P699T or L709P mutations using CRISPR/Cas9-mediated genome editing (*SI Appendix*, Fig. S1*A*). Two independent founder lines were established and maintained separately for each variant (*SI Appendix*, Fig. S1*B*); because both lines exhibited comparable phenotypes, data were pooled for analysis.

### The L709P Variant, But Not P699T, Causes Neonatal Lethality in Mice.

Unexpectedly, while *Sel1L^P699T^* mice were recovered at ~25% of expected Mendelian ratios at weaning, no homozygous *Sel1L^L709P^* mice were recovered among > 300 genotyped pups (Fig. 1*C*). Yet, homozygous *Sel1L^L709P^* pups were present at near-Mendelian frequencies (~17%) at postnatal day 0 (P0), indicating that lethality occurred postnatally. Although grossly normal at birth (*SI Appendix*, Fig. S1*C*), all *Sel1L^L709P^* pups died within 30 h, with over half dying in the first 3 h (Fig. 1*D*). Heterozygous *Sel1L^L709P/+^* mice were born and survived to weaning at normal Mendelian ratios (Fig. 1 *C* and *D*). At birth, body weights were indistinguishable across genotypes (Fig. 1*E*). On the other hand, *Sel1L^S658P^* KI mice exhibited partial neonatal survival, with ~50% dying after 30 h of birth (Fig. 1 *C* and *D*). Histological examination of major organs—including pancreas, liver, brain, and heart—revealed no overt developmental defects in *Sel1L^L709P^* neonates (Fig. 1 *F* and *G* and *SI Appendix*, Fig. S2). Together, these data demonstrate that L709P, but not P699T, causes neonatal lethality and represents a phenotype more severe than S658P.

### SEL1L L709P Impairs ERAD Function In Vivo and In Vitro.

**Fig. 2.**
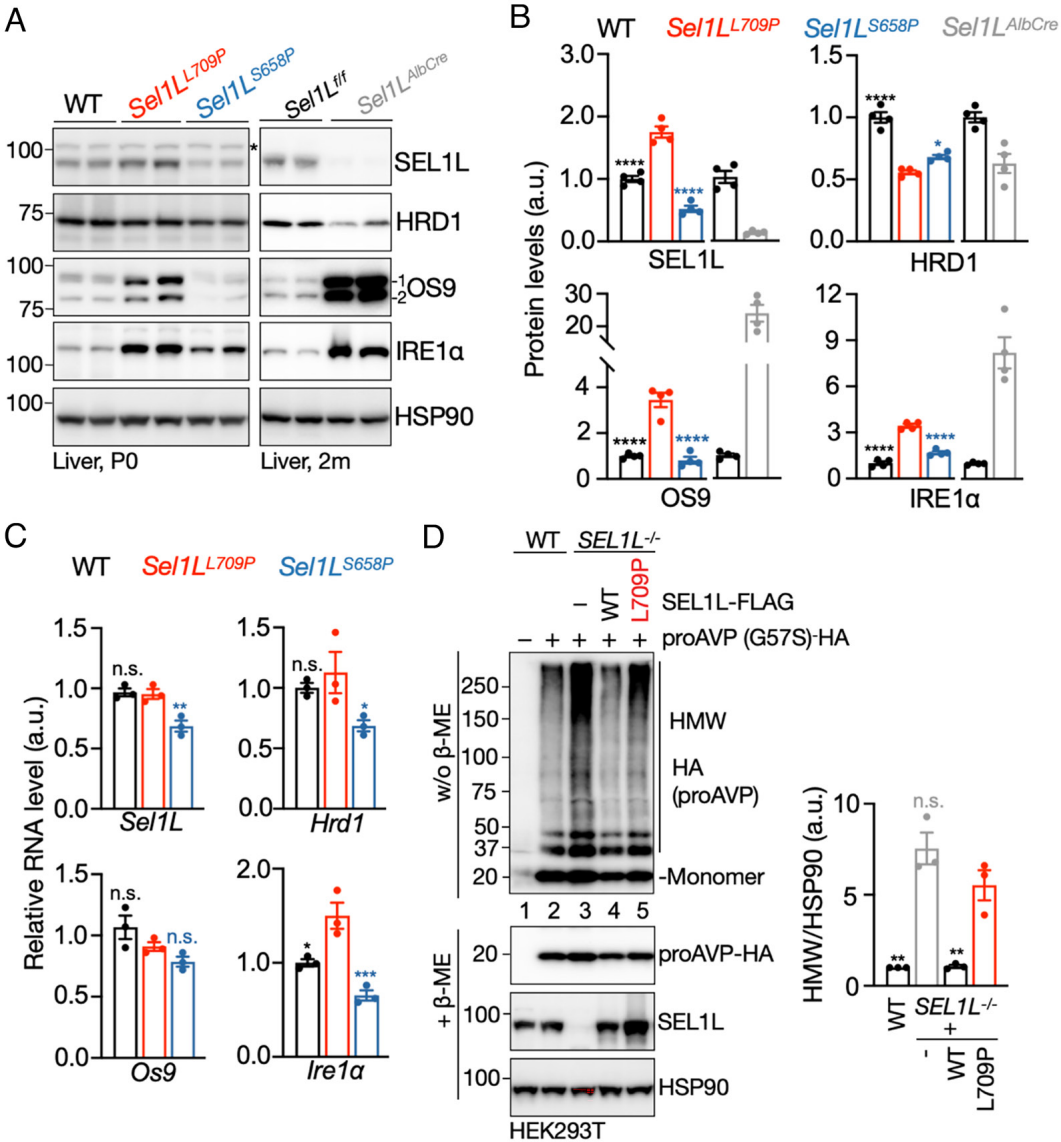
SEL1L L709P mutation impairs ERAD function in vivo and in vitro. (*A* and *B*) Immunoblots of core ERAD components (SEL1L, HRD1, OS9) and the endogenous substrate IRE1α in livers from P0 WT, *Sel1L^L709P^*, and *Sel1L^S658P^* KI mice, and 2-mo-old *Sel1L^f/f^* and *Sel1L^AlbCre^* mice (*A*), with quantification of protein levels shown in (*B*), normalized to the loading control HSP90. Asterisks, nonspecific bands. n = 4 mice per group. (*C*) qPCR analyses of ERAD-related gene expression in P0 livers, normalized to *L32*. n = 3 mice per group. (*D*) Reducing and nonreducing SDS-PAGE followed by immunoblotting to detect high-molecular-weight (HMW) aggregates of proAVP-G57S in *SEL1L^−/−^* HEK293T cells transfected with the indicated SEL1L-FLAG constructs. Quantification of HMW proAVP normalized to the loading control HSP90 shown on the *Right*. n = 3 independent samples. Values are mean ± SEM. Statistical comparisons are made relative to *SEL1L^L709P^* KI. n.s., not significant; *P < 0.05; **P < 0.01; ***P < 0.001; ****P < 0.0001 by one-way ANOVA with Dunnett’s multiple comparisons test.

To assess how L709P affects ERAD, we examined core ERAD components (SEL1L, HRD1, and OS9) across the three SEL1L KI models (L709P, S658P, P699T), using adult hepatocyte-specific Sel1L knockout mice (*Sel1L^AlbCre^*) as a reference for complete ERAD loss. In P0 livers, SEL1L protein was elevated ~ 1.6-fold in *Sel1L^L709P^* mice but reduced by >50% in *Sel1L^S658P^* mice (Fig. 2 *A* and *B*). Despite elevated SEL1L expression, HRD1 abundance decreased by ~45% in *Sel1L^L709P^* livers, similar to the reduction observed in *Sel1L^S658P^* livers.

In contrast, both isoforms of OS9 (OS9.1 and OS9.2) increased ~threefold in *Sel1L^L709P^* but remained unchanged in *Sel1L^S658P^* livers (Fig. 2 *A* and *B*), despite no changes in *Os9* mRNA levels (Fig. 2*C*). This accumulation aligns with prior evidence that OS9 may undergo codegradation with ERAD substrates via the SEL1L–HRD1 pathway (18, 28, 36, 44, 54). As expected with ERAD impairment, the ERAD substrate IRE1α (20) accumulated ~3.5-fold in *Sel1L^L709P^* and ~1.7-fold in *Sel1L^S658P^* livers (Fig. 2 *A* and *B*), accompanied by a modest (~40%) induction of *Ire1a* mRNA only in *Sel1L^L709P^* (Fig. 2*C*). These changes were milder than those in *Sel1L^AlbCre^* mice, where SEL1L was largely absent and HRD1 was reduced by 50% (Fig. 2 *A* and *B*).

Similar ERAD defects appeared in P0 *Sel1L^L709P^* brain, whereas *Sel1L^P699T^* brains resembled WT controls (*SI Appendix*, Fig. S3 *A* and *B*). We observed similar patterns in MEFs from the KI lines: *Sel1L^L709P^* cells showed elevated SEL1L, ~40% reduced HRD1, and ~twofold increases in OS9 and IRE1α—intermediate between WT-like *Sel1L^P699T^* and inducible *Sel1L^ERCre^* KO MEFs (18) (*SI Appendix*, Fig. S3 *C*–*E*).

To directly test ERAD activity, we evaluated degradation of the model substrate proAVP-G57S, which forms disulfide-linked high-molecular-weight (HMW) aggregates when SEL1L–HRD1 ERAD is impaired (55). Overexpression of WT SEL1L efficiently resolved these aggregates, whereas SEL1L L709P did not (lanes 3 to 5, Fig. 2*D*), confirming defective—but not completely abolished—ERAD function. Together, the L709P, S658P, and P699T KI models reveal a clear graded impairment of ERAD: L709P produces the most severe defect despite preserved SEL1L protein levels, placing it functionally between partial (S658P) and complete (*Sel1L* KO) ERAD loss.

### SEL1L L709P Expression Is Associated with Subtle Unfolded Protein Response (UPR) activation.

**Fig. 3.**
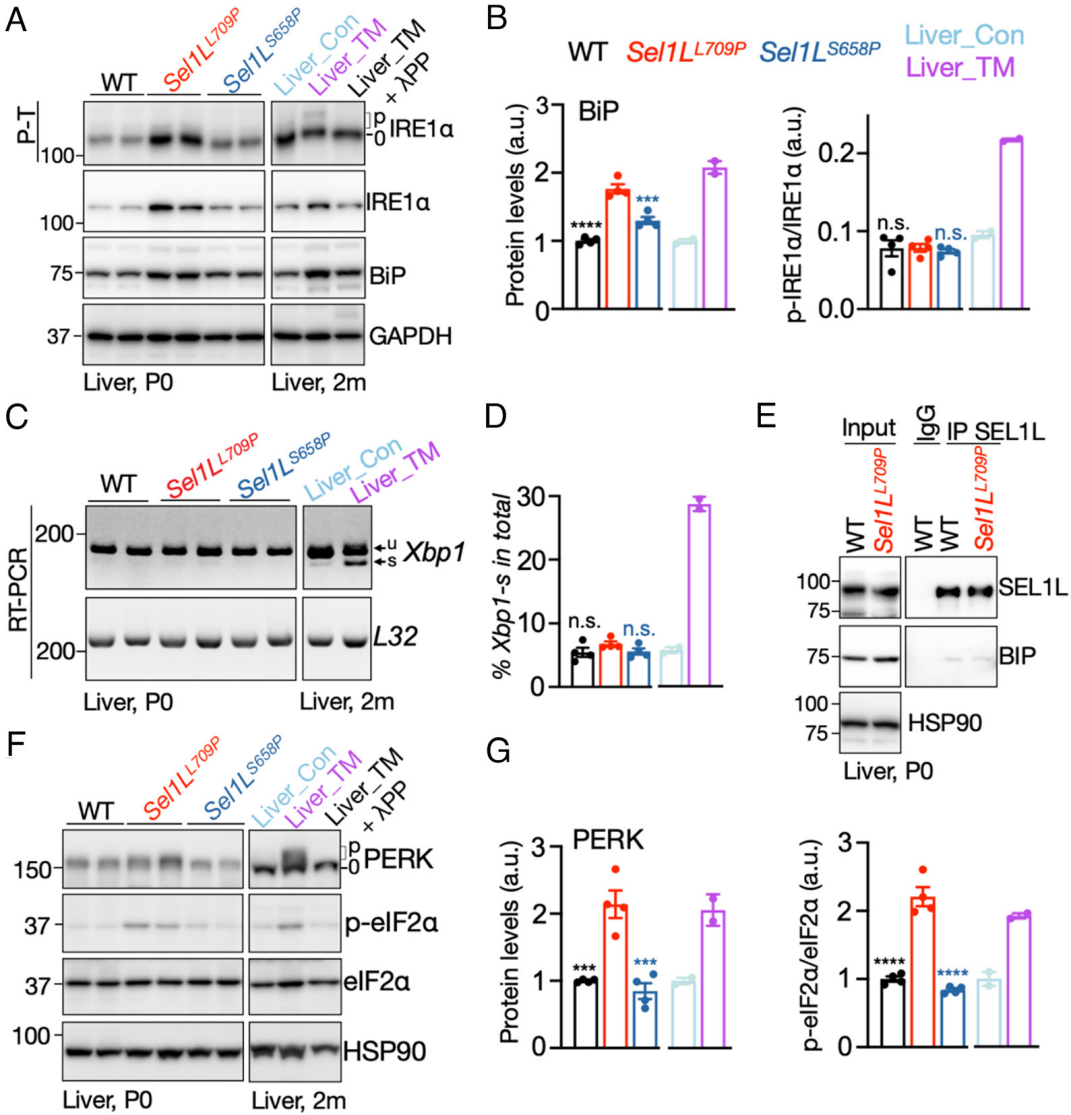
SEL1L L709P mutationis associated with mild ER stress in vivo. (*A* and *B*) Phos-tag gel analyses of phosphorylated IRE1α in liver tissues from P0 WT, *Sel1L^L709P^*, and *Sel1L^S658P^* KI mice. Livers from mice injected with PBS (CON) or tunicamycin (TM) were included as controls (A). Lamda phosphatase (λPP) treatment of the lysates was used to show protein phosphorylation. Total IRE1α and BiP protein levels were assessed using the standard SDS-PAGE. Quantification of BiP protein level normalized to GAPDH and the ratio of p-IRE1α in total IRE1α shown in (*B*). n = 4 mice per group. (*C* and *D*) RT-PCR analysis of *Xbp1* mRNA splicing in P0 and control livers (as in *A*) with quantification of percent of *Xbp1s* mRNA in total *Xbp1* mRNA shown in (*D*). n = 4 mice per group. (*E*) Coimmunoprecipitation (Co-IP) of endogenous SEL1L from P0 livers, followed by immunoblotting. HSP90, loading control. n = 3 mice per group. (*F* and *G*) Immunoblot analyses of PERK, total and phosphorylated eIF2α (p-eIF2α) in P0 and control livers (as in *A*), with quantification of protein level of PERK normalized to HSP90 and the ratio of p-eIF2α in total eIF2α shown in (*G*). n = 3 mice per group. Values are shown as mean ± SEM. Statistical comparisons are made relative to *SEL1L^L709P^*. n.s., not significant; *P < 0.05; **P < 0.01; ***P < 0.001; ****P < 0.0001 using one-way ANOVA with Dunnett’s multiple comparisons test.

In vivo, loss of SEL1L–HRD1 ERAD is known to elicit low-grade UPR activation through adaptative mechanisms such as ER dilation, induction of ER chaperones, and enhanced ER-phagy (12, 14, 56). To determine whether the *Sel1L^L709P^* mutation triggers similar responses, we examined two key UPR branches—IRE1α and PERK—in P0 livers. In *Sel1L^L709P^* livers, total IRE1α protein levels increased ~3.5-fold (Fig. 2 *A* and *B*), whereas the ratio of phosphorylated to total IRE1α remained unchanged (Fig. 3 *A* and *B*). Consistently, *Xbp1* mRNA splicing, a canonical readout of IRE1α activation, was largely unaltered (Fig. 3 *C* and *D*). BiP protein levels was moderately elevated (~1.7-fold) (Fig. 3 *A* and *B*), but the association between SEL1L and BiP (57) did not increase (Fig. 3*E*). For the PERK pathway, we observed a modest increase in PERK protein abundance accompanied by ~twofold higher phosphorylation of its downstream effector eIF2α (Fig. 3 *F* and *G*). Notably, the increases in BiP, PERK, and eIF2α phosphorylation were consistently greater in *Sel1L^L709P^* livers than in *Sel1L^S658P^* livers (Fig. 3 *A*, *B*, *F*, and *G*), suggesting a more pronounced yet still modest UPR.

**Fig. 4.**
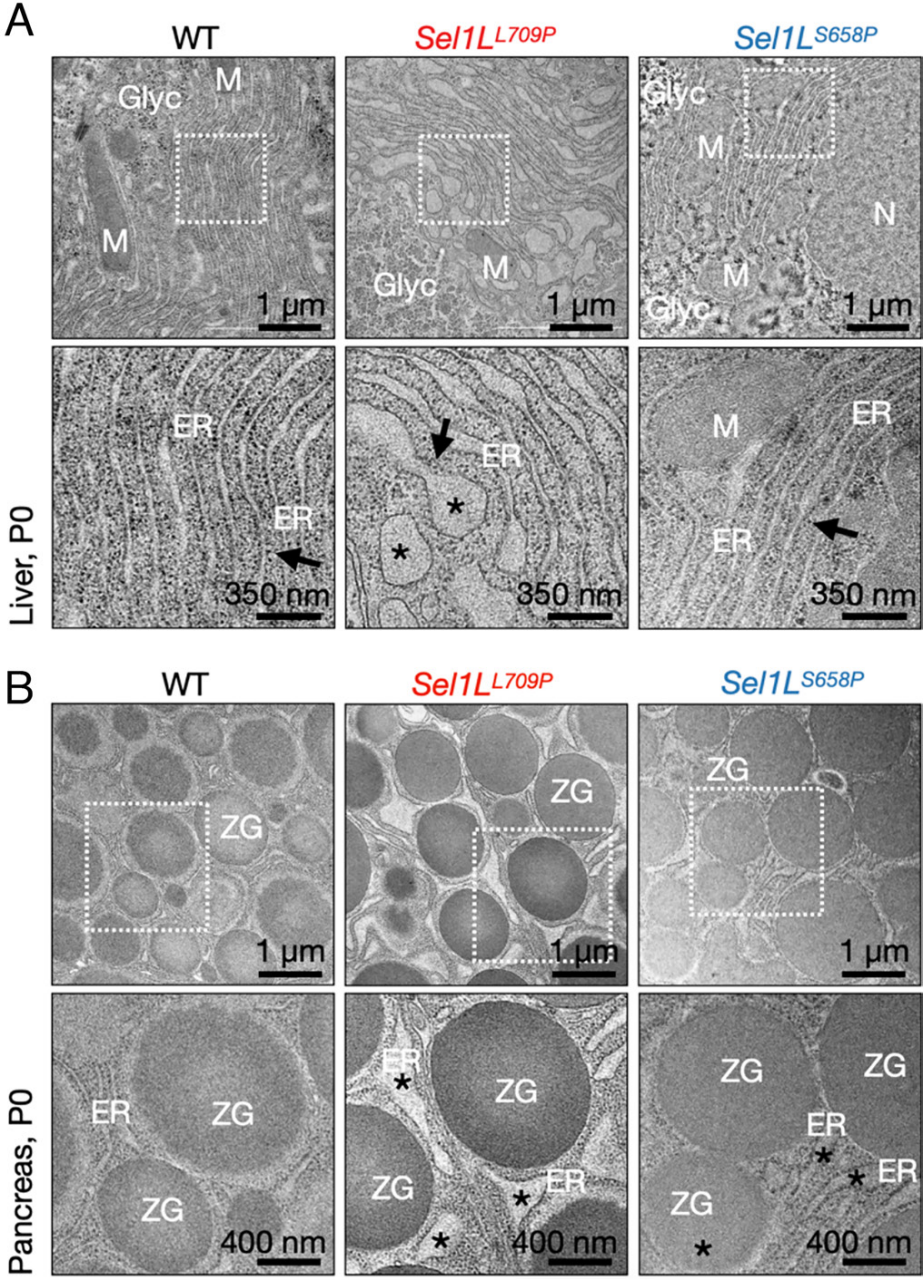
SEL1L L709P mutation causes mild ER dilation in hepatocytes and pancreatic acinar cells. (*A*) Representative TEM images of hepatocytes from P0 WT, *Sel1L^L709P^*, and *Sel1L^S658P^* livers. n = 3 mice with 30 to 40 cells per mouse. (*B*) Representative TEM images of P0 pancreatic acinar cells. n = 3 mice with 20 to 30 cells per mouse. N, nucleus; M, mitochondria; ER, endoplasmic reticulum (arrows); ZG, zymogen granules; Glyc, glycogen. Asterisk, dilated ER.

Transmission electron microscopy (TEM) corroborated these biochemical findings of mild ER stress. Hepatocytes from WT, *Sel1L^L709P^*, and *Sel1L^S658P^* mice maintained sheet-like ER morphology with abundant ribosomes (arrows); however, mild ER dilation was only evident in *Sel1L^L709P^* cells (asterisk, Fig. 4*A*). In pancreatic acinar cells, zymogen granules were preserved, but ER cisternae were swollen only in *Sel1L^L709P^* mice (asterisk, Fig. 4*B*). Together, these findings support that SEL1L L709P induces a subtle, adaptive UPR in vivo, a phenotype that is less pronounced in *Sel1L^S658P^* mice.

### SEL1L L709P Abolishes the SEL1L–HRD1 Interaction.

**Fig. 5.**
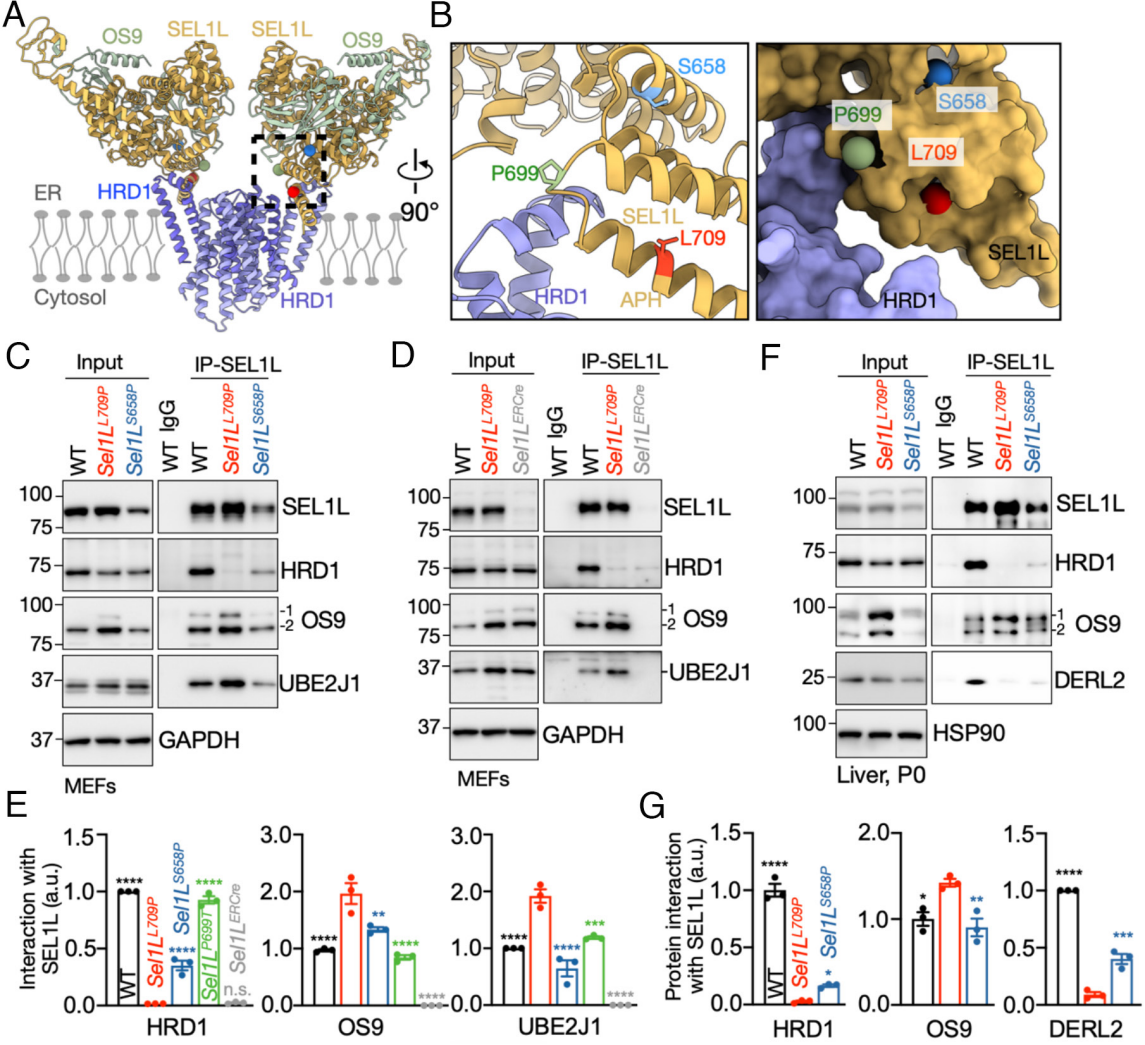
SEL1L L709P mutation abolishes SEL1L–HRD1 interaction in vivo and in vitro. (*A* and *B*) Dimeric OS9–SEL1L–HRD1 complex structure. A close-up view highlights the SEL1L–HRD1 interface with the location of three variants shown (*B*). APH, amphipathic helix of SEL1L. (*C*–*E*) Coimmunoprecipitation (Co-IP) of endogenous SEL1L from MEFs derived from WT, *Sel1L^S658P^*, *Sel1L^L709P^*, and *Sel1L^ERCre^*mice, followed by immunoblotting for ERAD components (*C* and *D*), with quantification shown in (*E*). GAPDH, a loading control. n = 3 mice per group. (*F* and *G*) Co-IP of endogenous SEL1L from livers of P0 neonates (WT, *Sel1L^S658P^*, and *Sel1L^L709P^*), followed by Western blot analyses of ERAD components (*F*), with quantification shown in (*G*). HSP90, a loading control. n = 3 mice per group. Values, mean ± SEM. Statistical comparisons are made relative to *SEL1L^L709P^*. n.s., not significant; *P < 0.05; **P < 0.01; ***P < 0.001; ****P < 0.0001 using one-way ANOVA with Dunnett’s multiple comparisons test.

Our recent cryo-EM structure of the mammalian OS9-SEL1L–HRD1 complex revealed a dimeric architecture (Fig. 5*A*) that differs markedly from its yeast counterpart (58–60). Within this structure, L709 resides in the SEL1L amphipathic helix (APH) and directly contacts the TM1-TM2 loop of HRD1, whereas P699 is positioned in the adjacent loop, and S658 resides in a nearby helix that does not directly participate in the interface (Fig. 5*B*). Structural modeling suggests that substituting L709 with proline disrupts the local helical geometry of the APH and destabilizes the SEL1L–HRD1 interface, whereas S658P may perturb the local helical stability of SEL1L without fully abolishing contact.

Coimmunoprecipitation of endogenous SEL1L in *Sel1L^L709P^* MEFs confirmed a near-complete loss of HRD1 binding—comparable to *Sel1L^ERCre^* KO cells—while interactions with OS9 and the E2 enzyme UBE2J1 (61) were preserved or mildly increased (Fig. 5 *C*–*E* and *SI Appendix*, Fig. S4*A*). This observation is in line with our recent observation that UBE2J1–HRD1 association requires SEL1L (44). In contrast, S658P reduced HRD1 binding by ~60% but retained normal OS9 binding and showed only a slight decrease in UBE2J1 association (Fig. 5 *C*–*E* and *SI Appendix*, Fig. S4*A*). The P699T variant behaved similarly to WT cells. Reciprocal HRD1 immunoprecipitation corroborated these findings, showing complete loss of SEL1L–HRD1 binding in *Sel1L^L709P^* MEFs and partial (~60%) reduction in S658P MEFs (*SI Appendix*, Fig. S4 *B* and *C*).

Consistent results were observed in P0 livers: the L709P substitution led to a near-total (~100%) loss of HRD1 association, compared with an ~80% reduction in the S658P mutant, while OS9 binding remained unaffected (Fig. 5 *F* and *G*). Interaction with the accessory component DERL2 (62) was similarly impaired, decreasing by ~90% in L709P and ~60% in S658P (Fig. 5 *F* and *G*). Together, these data demonstrate that L709 is a critical structural determinant of the SEL1L–HRD1 interface, and that its disruption functionally uncouples SEL1L from HRD1.

### SEL1L L709P Abolishes ERAD Complex Formation and Function in Human Cells.

**Fig. 6.**
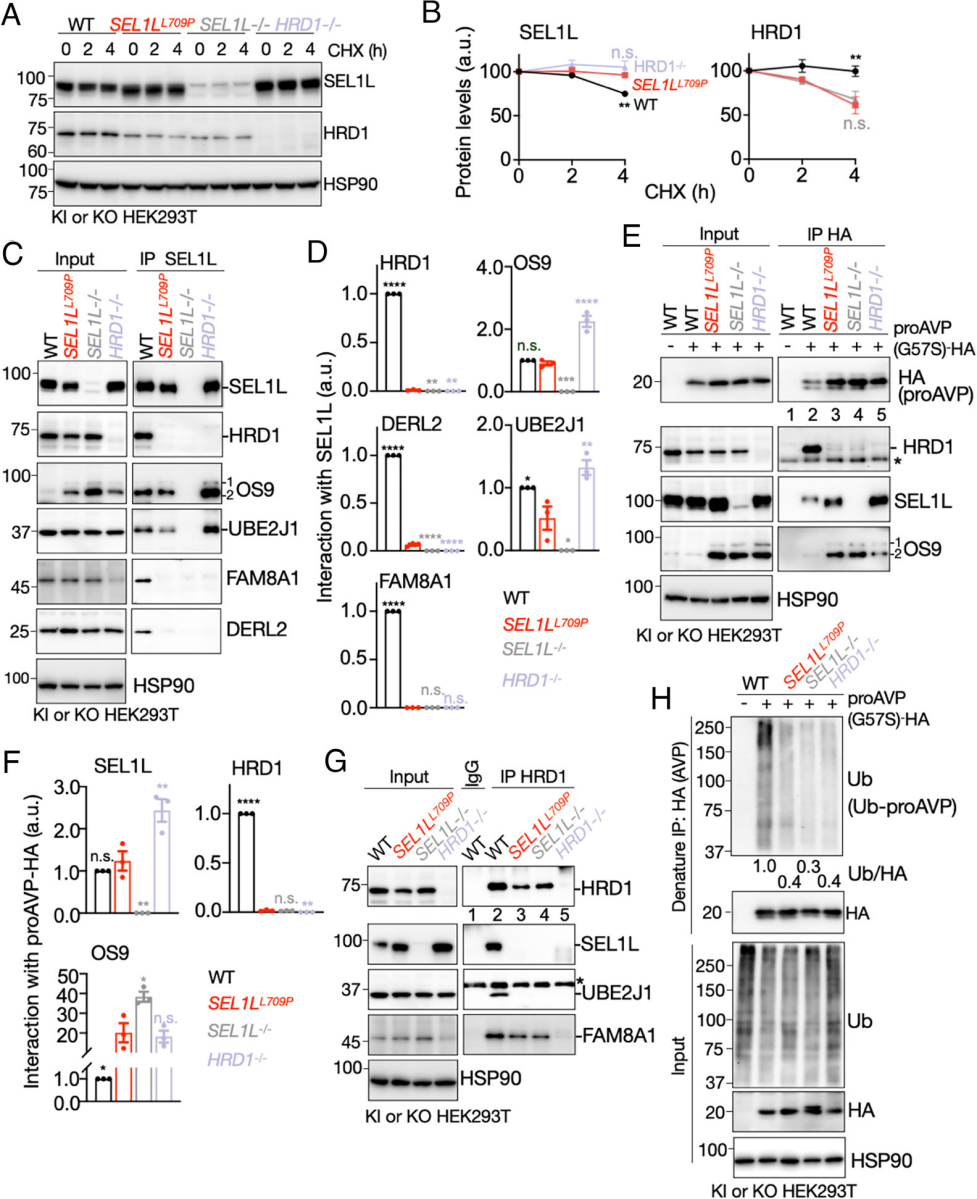
The SEL1L–HRD1 interaction is required for substrate engagement and E2 recruitment to HRD1 under basal conditions. (*A* and *B*) CHX translation shut-off assay in KI and KO HEK293T, with quantification normalized to HSP90 shown in (*B*). n = 3 independent samples. (*C* and *D*) IP of endogenous SEL1L in the indicated KI and KO HEK293T cells, followed by immunoblotting for ERAD components (*C*), with quantification normalized to SEL1L shown in (*D*); HSP90, a loading control. n = 3 independent samples. (*E* and *F*) IP of proAVP (G57S)-HA in transfected KI and KO HEK293T cells with quantification normalized to proAVP shown in (*F*). HSP90, loading control. The asterisk indicates a nonspecific band. n = 3 independent samples. (*G*) Coimmunoprecipitation of HRD1 in KI and KO HEK293T cells. HSP90, loading control. The asterisk indicates a nonspecific band. n = 3 independent samples. (*H*) Immunoblot of poly-ubiquitinated proAVP (G57S)-HA in transfected KO or KI HEK293T cells treated with 10 μM MG132 for 4 h. The quantification of AVP ubiquitination shown below the gel from three independent samples. HSP90, loading control. Values, mean ± SEM. Statistical comparisons are made relative to *SEL1L^L709P^*. n.s., not significant; *P < 0.05; **P < 0.01; ***P < 0.001; ****P < 0.0001 determined by simple linear regression (*B*) or by using one-way ANOVA with Dunnett’s multiple comparisons test (*D* and *F*).

To extend our findings to human cells, we generated biallelic *SEL1L^L709P^* KI HEK293T cells using CRISPR/Cas9 (*SI Appendix*, Fig. S5 *A* and *B*), with SEL1L- and HRD1-KO cells included as controls (44). As in mice, SEL1L protein abundance increased by ~30 to 40%, HRD1 levels decreased ~40%, and OS9 levels rose approximate sixfold (*SI Appendix*, Fig. S5 *C* and *D*). Canonical ERAD substrates, including IRE1α and CD147, were elevated by ~50 to 80%, phenocopying the *SEL1L* or *HRD1* KO cells (*SI Appendix*, Fig. S5 *C* and *D*). Cycloheximide translation shut-off assay showed that SEL1L was more stable and HRD1 was less stable in *SEL1L^L709P^* KI cells, indicating that the altered abundances primarily reflect changes in protein turnover (Fig. 6 *A* and *B*). Immunoprecipitation of endogenous SEL1L further revealed near-complete loss of HRD1, FAM8A1, and DERL2 association in *SEL1L^L709P^* KI cells, whereas interactions with OS9 and the E2 enzyme UBE2J1 remained intact (Fig. 6 *C* and *D*). These results confirm that L709P selectively disrupts ERAD complex assembly in human cells.

To determine the functional consequences for ERAD, we examined interaction with proAVP-G57S-HA in WT, KI, and KO HEK293T cells. In *SEL1L^L709P^* KI HEK293T cells, proAVP associated with OS9 and SEL1L but failed to engage HRD1 (lanes 3 to 5, Fig. 6 *E* and *F*), demonstrating defective substrate transfer to the ligase. A similar defect was observed for another ERAD substrate, POMC-C28F (63) (*SI Appendix*, Fig. S6 *A* and *B*). Reciprocal HRD1 immunoprecipitation further showed that L709P abolished recruitment not only of SEL1L but also of the E2 enzyme UBE2J1 (Fig. 6*G*). Consequently, polyubiquitination of proAVP and POMC was severely impaired, phenocopying *SEL1L^−/−^* and *HRD1^−/−^* cells (Fig. 6*H* and *SI Appendix*, Fig. S6C). Together, these findings demonstrate that the L709P mutation disrupts ERAD by disconnecting both substrate delivery and E2 enzyme recruitment from HRD1.

### HRD1 Overexpression Partially Rescues ERAD Defects in *Sel1L*-Deficient Cells.

Inspired by yeast studies in which Hrd1 overexpression can bypass the requirement of Hrd3 (42, 46, 47), we asked whether elevated HRD1 expression could similarly compensate for SEL1L dysfunction in mammalian cells. In *SEL1L^L709P^* KI and *SEL1L^−/−^* HEK293T cells, HRD1 overexpression restored the degradation of the endogenous ERAD substrates CD147 (64) and integrin αV (ITGAV) (28) in a dose-dependent manner, showing decay kinetics similar to those observed in WT and *HRD1^−/−^* HEK293T cells transfected with HRD1 (*SI Appendix*, Fig. S7 *A* and *B*). These results suggest that while SEL1L is essential for efficient HRD1-mediated ERAD under physiological conditions, supraphysiological HRD1 levels can partially bypass the requirement for SEL1L and restore substrate processing in mammalian cells.

## Discussion

**Fig. 7.**
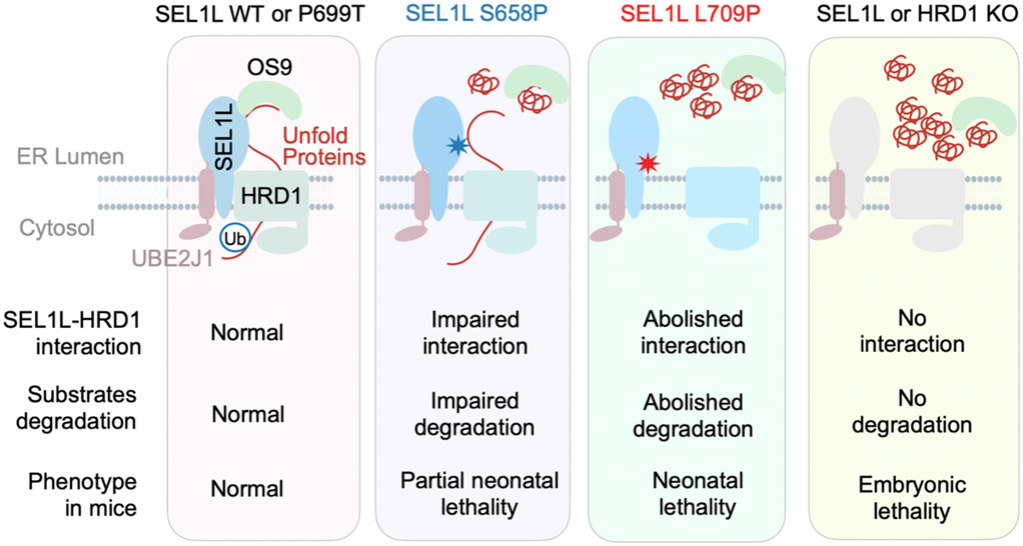
Model illustrating the functional and physiological roles of SEL1L–HRD1 coupling. Our data reveal a coherent structure–function–physiology relationship: the extent of SEL1L–HRD1 disruption corresponds directly to ERAD impairment and stratifies organismal survival.

Although SEL1L and HRD1 are recognized as core components of mammalian ERAD, whether SEL1L is directly required for HRD1 function in vivo has remained unresolved because tools to selectively disrupt their interaction were lacking. By generating three SEL1L knock-in variants that span intact (P699T), partially disrupted (S658P), and abolished (L709P) SEL1L–HRD1 binding, we define a graded series of ERAD dysfunction with corresponding physiological outcomes (Fig. 7). This allelic series reveals a quantitative relationship between the strength of the SEL1L–HRD1 interface, ERAD capacity, and postnatal viability: P699T preserves ERAD and viability; S658P yields partial ERAD defects and incomplete neonatal survival; and L709P eliminates SEL1L–HRD1 coupling, produces severe ERAD failure, and results in uniform neonatal lethality. These findings identify SEL1L–HRD1 interaction as a physiologically indispensable node in mammalian ERAD and establish a conceptual and structural framework for understanding—and ultimately targeting using small molecules—SEL1L–HRD1 ERAD in human disease.

Our first major insight is that SEL1L–HRD1 interaction is directly required for HRD1 activity under basal conditions. Disrupting the SEL1L–HRD1 interface impairs two fundamental steps of ERAD: substrate engagement via lectins and recruitment of the E2 enzyme UBE2J1 to HRD1. Unlike in yeast, where Hrd3 loss does not abolish substrate association with Hrd1 (47), mammalian HRD1 requires SEL1L to coordinate both substrate delivery and ubiquitination. These mechanistic findings support a model in which SEL1L functions not only as a scaffold but as an organizing center that couples substrate recognition with the ubiquitination machinery. Notably, supraphysiological HRD1 expression partially bypasses SEL1L dependence, suggesting that under certain stress or disease contexts, mammalian cells may adopt compensatory strategies analogous to those described in yeast. Whether such bypass pathways operate in specific tissues or developmental windows remains an open question.

A second major insight is the dose-dependent relationship between ERAD impairment and organismal survival. Complete loss of *Sel1L* or *Hrd1* causes embryonic lethality, whereas acute adult deletion leads to rapid mortality (15–18). Here, we show that selectively eliminating SEL1L–HRD1 coupling through the L709P mutation results in neonatal death despite preserved SEL1L abundance and ~50% HRD1. In contrast, the hypomorphic S658P allele supports partial neonatal survival. This graded relationship mirrors clinical observations in ERAD-associated neurodevelopmental disorders, where disease severity inversely correlates with residual ERAD activity in patients with hypomorphic SEL1L or HRD1 variants (36, 37, 52). Together, these data strengthen the emerging view that ERAD dysfunction constitutes a dosage-sensitive axis underlying human developmental and neurological disease.

The specific cause of neonatal lethality in *SEL1L^L709P^* mice remains unresolved. Major organ histology appears normal, and ultrastructural changes are limited to mild ER dilation. Consistent with prior studies, disruption of SEL1L–HRD1 ERAD triggers only a modest UPR, likely due to compensatory buffering mechanisms such as chaperone upregulation or autophagy activation (12–14, 52, 65). Because neonatal survival depends on precise execution of respiration, thermoregulation, and nutrient acquisition (66), future studies will be needed to determine whether loss of the SEL1L–HRD1 interface compromises these processes and its underlying mechanisms.

## Materials and Methods

### Genetic Variants.

Human SEL1L variants (P699T and L709P) were identified through a search of clinical exome and genome sequencing database from Baylor Genetics Laboratory. The full study protocols and protocols for written, informed consent were approved by the Institutional Review Board for Health Sciences Research (IRB-HSR, University of Virginia, HSR230351).

### Mouse Models.

*Sel1L^S658P^* KI mice (44) and *Sel1L^AlbCre^* mice (22, 67, 68) were previously generated. *Sel1L^P699T^* and *Sel1L^L709P^* KI mice (corresponding to SEL1L P695T and L705P in mice, respectively) were generated on the B6/SJL background via CRISPR/Cas9 at the University of Michigan Molecular Genetics Core. Two single-guide RNAs (sgRNAs) targeting exon 20 of the Sel1L gene were designed using CRISPOR (https://crispor.gi.ucsc.edu/) (sgRNA1: CTAGGACATTCACCTTGCAA; sgRNA2: AAGCAAACGTAAGTGAGCCG), synthesized via the Synthego sgRNA Synthesis Kit, and validated in fertilized mouse oocytes. The donor template (Ultramer dsDNA, IDT) contained the desired mutation and silent mutations to facilitate homology-directed repair:

Donor 1 (P695T): ACACAAATTTTTCTGAAACATTTCCTTTGCTCTCTGTCCTCTAGGATATTCATCTTGCTAAACGCTTTTATGACATGGCAGCCGAAGCTAGC**ACA**GATGCACAAGTACCTGTGTTCCTCGCACTCTGCAAATTAGGTGTCGTCTATTTCTTACAGTACATACGGGAGGCCAATGTAAGTGAGCCGTTGCTCTTAGTTACA;

Donor 2 (L705P): ACACAAATTTTTCTGAAACATTTCCTTTGCTCTCTGTCCTCTAGGATATTCATCTTGCTAAACGCTTTTATGACATGGCAGCCGAAGCTAGCCCAGATGCACAAGTACCTGTGTTCCTCGCA**CCC**TGCAAATTAGGTGTCGTCTATTTCTTACAGTACATACGGGAGGCCAATGTAAGTGAGCCGTTGCTCTTAGTTACA

This template was coinjected at final concentrations of 100 ng/µL Cas9 protein and 50 ng/µL sgRNA in injection buffer (10 mM Tris-HCl, 0.1 mM EDTA, pH 7.5). Approximately 200 fertilized B6/SJL zygotes were injected and transferred into pseudopregnant CD-1 females. Founder mice were genotyped by Sanger sequencing of PCR amplicons spanning exon 20 (F: 5′-TTAGGTCGGATCTTGAAAGGCTAAC-3′; R: 5′-CACAGGTTACCCACGAGGAATTC-3′) and backcrossed to C57BL/6J for three generations prior to generating homozygous KI lines and littermate controls.

All mouse strains used in this study were on a C57BL/6J genetic background. Mice were housed in a specific pathogen-free facility at 22 ± 1 °C under a 12-h light/dark cycle with 40 to 60% humidity and fed a low-fat diet (13% fat, 57% carbohydrate, 30% protein; LabDiet 5LOD). All animal procedures were approved by the University of Virginia (Protocol #4459) and were conducted in accordance with the guidelines of the NIH.

### Neonatal Survival Curve Analysis.

To assess neonatal survival, pups were monitored from birth (postnatal day 0, P0) through the first 48 h of life. Litters were inspected at every 3 h, and the number of surviving pups was recorded at each designated time point. Dead pups were removed at each inspection, and tail biopsies were collected immediately for genotyping. After 48 h, tail samples were collected from all surviving pups. Pups exhibiting spontaneous movement and active breathing were considered alive. Genotyping was performed on tail biopsies collected from both surviving and nonsurviving pups. Kaplan–Meier survival curves were generated using GraphPad Prism software, with survival plotted as the percentage of live pups over time. Statistical significance between genotypes was determined using the log-rank (Mantel–Cox) test.

### Histological Analysis.

P0 mice were fixed following a modified protocol based on the Mouse Phenotyping Core at UCSD (https://mousepheno.ucsd.edu/histo.shtml). Neonatal pups were killed and immediately immersion-fixed. A small midline abdominal incision was made to improve fixative penetration, and pups were submerged in 30 mL of freshly prepared fixative (ethanol:37% formaldehyde:glacial acetic acid, 6:3:1 v/v) at 4 °C for 72 h, with the solution replaced every 24 h. To ensure complete fixation, tissues were then sagittally sectioned and postfixed in 10% neutral-buffered formalin (Sigma HT501320) for an additional 3 d before processing. Fixed tissues were paraffin-embedded, sectioned, and H&E-stained by the UVA Research Histology Core.

### Generation of MEFs.

Inducible *Sel1L*-deficient MEFs (*Sel1L^ERCre^*) were generated as previously described (18) by treating *Sel1L^f/f^;ERCre^+^* MEFs with 400 nM 4-OHT (Sigma, H7904) for 3 d. MEFs were generated from embryonic day 15.5 (E15.5; counting noon of the day when the vaginal plug was found as E0.5) embryos obtained from matings between heterozygous mice carrying different SEL1L variants (P699T, S658P, or L709P) as previously described (18). Briefly, the head and liver were removed from each embryo (and retained for genotyping), and the remaining tissue was finely minced and digested in 5 mL of trypsin at 37 °C for 10 min. The digested tissue was then pipetted vigorously to obtain a single-cell suspension and plated in 10-cm culture dishes. MEFs were seeded at ~1×10^6^ cells per 10 cm dish and cultured in DMEM (Gibco) supplemented with 10% fetal bovine serum (FBS; Fisher Scientific, FB12999102) at 37 °C in a humidified incubator with 5% CO_2_ and expanded for experiments.

### CRISPR/Cas9-Mediated Gene KO and KI in Cells.

HEK293T cells (ATCC) were cultured in DMEM (Gibco, 11965118) supplemented with 10% fetal bovine serum (FBS; Fisher Scientific) at 37 °C in a humidified incubator with 5% CO_2_. SEL1L and HRD1 KO HEK293T cell lines were generated as previously described (44). Briefly, Sel1L and Hrd1 guide RNAs were cloned into lentiCRISPR v2 (Addgene #52961). HEK293T cells (~70% confluence in 6-well plates) were transfected with 2 µg plasmid DNA per well using polyethyleneimine (PEI; 5 µL per µg DNA). After 24 h of puromycin selection (2 µg/mL), surviving cells were single-cell cloned in 96-well plates and expanded. KO efficiency was confirmed by Western blot using anti-SEL1L (homemade, 1:10,000) and anti-HRD1 (Proteintech #13473-1-AP, 1:2,000) antibodies.

Guide RNAs:

*SEL1L*,

F: 5′-CACCGGGCTGAACAGGGCTATGAAG-3′;

R: 5′-AAAC CTTCATAGCCCTGTTCAGCCC-3′

*HRD1*,

F: 5′-CACCGGGCCAGCCTGGCGCTGACCG-3′;

R: 5′-AAACCGGTCAGCGCCAGGCTGGCCC-3′

SEL1L L709P KI HEK293T cells were generated via CRISPR-Cas9-Homology-Directed Repair (CIRSPR-Cas9-HDR) as previously described (44). Briefly, crRNA (IDT) encoding the gRNA sequence was mixed with Alt-R tracrRNA (IDT) and annealed by heating and cooling at room temperature. The duplex was then incubated with Alt-R Cas9 enzyme (IDT) to form the RNP complex. The RNP, HDR donor oligo (IDT), and Alt-R Cas9 Electroporation Enhancer (IDT) were combined with HEK293T cell suspension in Electroporation Solution (Ingenio) and electroporated using a Lonza Nucleofector IIb. Electroporated cells were transferred to DMEM with 10% FBS and Alt-R HDR Enhancer V2 (IDT) and cultured at 37 °C with 5% CO_2_. After 5 d, genomic DNA was extracted, the target region was PCR-amplified (HotStart Taq 2X Master Mix, Abclonal), and Sanger-sequenced (Eurofins) to assess editing efficiency. In parallel, cells were plated into 96-well plates for single-cell colonies. After 10 d, ~80 colonies were expanded in 24-well plates. The *SEL1L^L709P^* locus was PCR-amplified and sequenced, and homozygous clones were selected for further experiments.

crRNA (guide sequence): 5’- GACGACGCCCAATTTGCAGA -3’

HDR Donor Oligo (the desired mutation is underlined): 5’-

TCCCGTATGTACTGCAAGAAATAGACGACGCCCAATTTGCAGGGGGCTAGGAAGACTGGAACTTGTGCATCTGGGCTGGCTTC-3’

Amplification PCR primers:

F: 5’- AGCTTGGCATTTTTGTTTAGGTG -3’

R: 5’- CGTCAGGAAGTGTCAAACGCT -3’

Sequencing primer:

5’- TTTGACAAGAAATGCATTTTTG -3’

### Phos-tag SDS-PAGE.

IRE1α was resolved using Phos-tag SDS-PAGE (5% gel containing 50 μM Phos-tag and 50 μM MnCl_2_) as previously described (69, 70) with minor modifications. Briefly, gels were run at 90 V for 20 min and 100 V for 2 h at room temperature. Following electrophoresis, Phos-tag gels were incubated in 1 mM EDTA in transfer buffer for 10 min to remove Mn^2+^ ions, then equilibrated in standard transfer buffer for 10 min. Proteins were transferred to PVDF membranes at 108 V for 90 min. After transfer, membranes were blocked and probed as in conventional Western blotting.

### Drug Treatment.

MEFs or HEK293T cells were treated with 50 μg/mL cycloheximide (CHX) for the indicated time points or 10 μM MG132 for 4 h, followed by Western blot or immunoprecipitation. CHX (Sigma C4859) and MG132 (MedChem Express, HY-13259C) was prepared as a 10 mg/mL and 10 mM stocks in DMSO, respectively. DMSO (0.1%) was used as vehicle control in all experiments.

### Immunoprecipitation.

Cell and tissue lysates were prepared in lysis buffer (150 mM NaCl, 25 mM Tris-HCl pH 7.5, 0.2% NP-40, 0.1% Triton X-100, protease inhibitors), and lysates containing 1 mg total protein were incubated with 1 µg anti-SEL1L or anti-HRD1 antibodies overnight at 4 °C, followed by Protein A agarose (Invitrogen). Bound proteins were washed three times with lysis buffer (5 min each on a rotator at 4 °C), then eluted using 2× SDS sample buffer (prepared by diluting 5× SDS sample buffer with lysis buffer) at 95 °C for 5 min, and subsequently analyzed by SDS-PAGE and Western blotting. To detect DERL2, the bound proteins were eluted with 0.1 M glycine buffer (pH 2.5) at room temperature for 5 min, followed by neutralization with 0.2 volumes of 1 M Tris-HCl (pH 8.0). The neutralized eluates were then mixed with 1× SDS sample buffer for loading. For input samples, lysates were mixed with 1× SDS sample buffer and incubated at room temperature for 5 min before direct loading.

For ubiquitination assays, lysates from cells transfected with proAVP-G57S-HA and POMC-C28F-HA were prepared in denaturing buffer (150 mM NaCl, 50 mM Tris-HCl pH 7.5, 1 mM EDTA, 1% NP-40, 1% SDS, 5 mM DTT, protease inhibitors), diluted 10-fold in the former lysis buffer, and incubated with anti-HA agarose (Sigma A2095) overnight at 4 °C. Bound proteins were washed and eluted using the same conditions as described in the previous immunoprecipitation experiment, and subsequently analyzed by SDS-PAGE and Western blotting. The intensity of the coimmunoprecipitated (prey) band was normalized to the bait signal to quantify the binding efficiency.

### Nonreducing SDS-PAGE.

Cell lysates were prepared in NP-40 buffer (50 mM Tris-HCl pH 8.0, 0.5% NP-40, 150 mM NaCl, 5 mM MgCl_2_ and protease inhibitor) supplemented with 10 mM N-ethylmaleimide, incubated at 37 °C for 30 min, and resolved on 6 to 15% gradient gels under nonreducing conditions as previously described (68).

### TEM.

Mice were perfused with 4% glutaraldehyde and 4% paraformaldehyde in 0.1 M Sorenson’s buffer. Tissues were postfixed overnight in 3% glutaraldehyde and 3% paraformaldehyde in 0.1 M Sorenson’s buffer. Following fixation, tissues were embedded, sectioned, and stained with uranyl acetate and lead citrate by the University of Virginia Molecular Electron Microscopy Core. Images were acquired using a Tecnai F20 transmission electron microscope at the same facility.

### Statistical Analysis.

Each experiment was performed in at least three independent biological replicates. For animal studies, “n” represents individual animals; for cell-based assays, n represents independent samples. All statistical analyses were performed using GraphPad Prism 10.0. Results are presented as mean ± SEM. Statistical significance was determined using one-way ANOVA with post hoc Dunnett’s multiple comparisons test, log-rank (Mantel–Cox) tests for survival curve analyses, and simple linear regression for protein decay curves, as appropriate. A *P*-value < 0.05 was considered statistically significant.

## Supplementary Material

Appendix 01 (PDF)

## Data Availability

All study data are included in the article and/or *SI Appendix*.
