# Supplementary material for: Functional integrity of the SEL1L–HRD1 complex is critical for endoplasmic reticulum–associated degradation and organismal viability
Source: Proc Natl Acad Sci U S A. 2026 Feb 5;123(6):e2517927123. doi: 10.1073/pnas.2517927123 (PMC12891039; doi:10.1073/pnas.2517927123)
Supplement: Supplementary file 1 — Appendix 01 (PDF) [file pnas.2517927123.sapp.pdf]

PNAS

Supporting Information for

Functional integrity of the SEL1L-HRD1 complex is critical for endoplasmic reticulum-associated degradation and organismal viability

Xiawei Zhang[1], Liangguang Leo Lin[1] , Linxiu Pan[1], Xiaoqiong Wei[1], Huilun Helen Wang[1], Zexin Jason Li[1], and Ling Qi[1] *

* Correspondence: xvr2hm@virginia.edu

This PDF file includes:

Supporting text
SI References
Figures S1 to S7

Supporting Information Text

Methods

Genetic Analysis

Evolutionary conservation of these residues was assessed using Clustal W and visualized with Jalview (1). Orthologous protein sequences from human (NP_005056.3), chimpanzee (JAA44458.1), pig (XP_020955243), mouse (NP_001034178.1), Drosophila (NP_001262882.1), yeast (QHB10358.1), and chicken (XP_040558090.1) were aligned.

Mouse Genotyping

Genomic DNA from tail biopsies was amplified using the following primers:
WT-F 5′-CTGTCCTCTAGGACATTCACCTTGCA-3′;
WT-R 5′-CAGAAACCACCTGCACCGAT-3′;
KI-F 5′-CTGTCCTCTAGGATATTCATCTTGCT-3′;
KI-R 5′-CAGAAACCACCTGCACCGAT-3′).
L709P and P699T KI mice shared the same primer sets for genotyping. Genotyping procedure and primers for SEL1L S658P KI mice were previously described (2).

Plasmids and Transfection

Mouse *Sel1L* WT and S658P in the pcDNA3 expression vector with a C-terminal FLAG tag were previously reported (2). Point mutations (P699T, L709P, and S658P) of *Sel1L* were introduced by site-directed mutagenesis (2) and verified by Sanger sequencing. Primers to generate the mutants are:
P699T: F 5′-GGCAGCCGAAGCTAGCACAGATGCACAAG-3′,
R 5′-CTTGTGCATCTGTGCTAGCTTCGGCTGCC-3′)

L709P: F 5′-CCTGTGTTCCTCGCACCCTGCAAATTAGGTG-3′,
R 5′-CACCTAATTTGCAGGGTGCGAGGAACACAGG-3′,

For most experiments, HEK293T cells were seeded at a density of 1 × 10^6 cells per well in six-well plates or 3 × 10^6 cells per 6-cm dish. After 24 hours, the medium was replaced with fresh, pre-warmed DMEM, and cells were transfected using PEI at a ratio of 5 µL PEI per 1 µg plasmid DNA. Cells were harvested 24 hours later or subjected to drug treatments as indicated. For the transfection of SEL1L variants (*SEL1L-FLAG*, *SEL1L-L709P-FLAG*, *SEL1L-P699T-FLAG*, and *SEL1L-S658P-FLAG*), 1 µg of plasmid DNA was used per well (six-well plate) or 3 µg per 6-cm dish. For proAVP-G57S-HA (3) and POMC-C28F-HA (4) plasmid, 1 µg of plasmid DNA was used per well (six-well plate) or 3 µg per 6-cm dish. For transfection of the human HRD1-MYC plasmid, 0.2 µg and 0.5 µg per well (six-well plate) were used for Fig. S7A-C.

Immunoblotting

Mouse tissues and MEFs were lysed in Triton X-100 buffer (50 mM Tris-HCl pH 7.5, 150 mM NaCl, 1% Triton X-100, 1 mM EDTA) supplemented with protease/phosphatase inhibitors (Sigma). Lysates were cleared by centrifugation (16,000 g, 10 min), and protein concentrations of supernatants were measured using Bio-Rad Protein Assay Dye (Bio-Rad). A total of 20-50 µg of protein was denatured at 95°C for 5 minutes in 5x SDS sample buffer containing 250 mM Tris-

HCl (pH 6.8), 10% SDS, 0.05% bromophenol blue, 50% glycerol, and 1.44 M β-mercaptoethanol. SDS-PAGE and Western blotting were performed using standard protocols (5, 6). The blots were incubated in 2% BSA/Tri-buffered saline tween-20 (TBST) with primary antibodies overnight at 4°C: anti-HSP90 (Santa Cruz, #sc-13119, 1:5,000), anti-GAPDH (Proteintech, #60004-1, 1:5000), anti-SEL1L (home-made, 1:10,000) (7), anti-HRD1 (Proteintech, #13473-1, 1:2,000), anti-OS9 (Abcam, #ab109510, 1:5,000), anti-CD147 (Proteintech, #11989-1, 1:3,000), anti-IRE1α (Cell Signaling, #3294, 1:2,000), anti-UBE2J1 (Santa Cruz, #sc-377002, 1:3,000), anti-HA (Sigma, #2018, 1:1,000), anti-PERK (Cell Signaling, #3192, 1:5000), anti-eIF2α (Cell Signaling, #9722, 1:5000), anti-p-eIF2α (Cell Signaling, #9721, 1:1000) and anti-ubiquitin (Santa Cruz, #sc-8017, 1:1000), anti-DERL2 (gift from Chih-Chi Andrew Hu, 1:1000), anti-FAM8A1 (Proteintech, #24746-1-AP,1:3000), anti-ITGAV (Proteintech, #27096-1-AP, 1:2000). Membranes were washed with TBST and incubated with the appropriate HRP-conjugated secondary antibodies at room temperature for 1 hour prior to detection using an ECL chemiluminescence system (Bio-Rad). For general Western blot, HRP-conjugated secondary antibodies (Bio-Rad, 1:10,000) were used. For immunoprecipitation samples, either anti-Rabbit IgG TrueBlot HRP (Rockland, #18-8816-33, 1:500) or anti-Mouse IgG TrueBlot HRP (Rockland, #18-8817-31, 1:500) was used as appropriate. Band intensity was quantified using Image Lab software (Bio-Rad).

Protein lysates from the livers of 2-month-old mice treated with tunicamycin (TM, 1 mg/kg, i.p.) for 24 hours as a positive control for UPR. For phosphatase treatment, 100 µg of tissue lysate was incubated with 1 µL of lambda phosphatase (λPPase; New England BioLabs, catalog #P0753S) in 1× PMP buffer (New England BioLabs, catalog #B0761S) supplemented with 1 mM $MnCl_2$ (New England BioLabs, catalog #B1761S) at 30 °C for 60 minutes. The reaction was terminated by adding 5× SDS sample buffer and heating at 90 °C for 5 minutes, as previously described (8).

RNA Extraction, RT-PCR and Q-PCR

Total RNA was extracted using TRI Reagent (Sigma) as previously described (2). *Xbp1* mRNA splicing was analyzed by RT-PCR using intron-flanking primers (F: 5′-ACGAGGTTCCAGAGGTGGAG-3′; R: 5′-AAGAGGCAACAGTGTCAGAG-3′) as previously described (9). PCR products were resolved by agarose gel electrophoresis and quantified with Image Lab. For qPCR, gene expression levels were normalized to *L32*. Primer sequences were as follows:

L32 F: 5'-GAGCAACAAGAAAACCAAGCA-3'; R: 5'-TGCACACAAGCCATCTACTCA-3'.
Sel1L F: 5'-TGGGTTTTCTCTCTCTCCTCTG-3'; R: 5'-CCTTTGTTCCGGTTACTTCTTG-3'.
OS9 F: 5'-GCTGGCTGACTGATGAGGAT-3'; R: 5'-CGGTAGTTGCTCTCCAGCTC-3'.
Hrd1 F: 5'-AGCTACTTCAGTGAACCCCACT-3'; R: 5'-CTCCTCTACAATGCCCACTGAC-3'.
Ire1α F: 5'-ATCTGCGCAAATTCAGAACC-3'; R: 5'-CTCCATGGCTTGGTAGGTGT-3'.

References

1. A. M. Waterhouse, J. B. Procter, D. M. Martin, M. Clamp, G. J. Barton, Jalview Version 2--a multiple sequence alignment editor and analysis workbench. *Bioinformatics* **25**, 1189-1191 (2009).
2. L. L. Lin *et al.*, SEL1L-HRD1 interaction is required to form a functional HRD1 ERAD complex. *Nature communications* **15**, 1440 (2024).
3. G. Shi *et al.*, ER-associated degradation is required for vasopressin prohormone processing and systemic water homeostasis. *J Clin Invest* **127**, 3897-3912 (2017).
4. G. H. Kim *et al.*, Hypothalamic ER-associated degradation regulates POMC maturation, feeding, and age-associated obesity. *J Clin Invest* **128**, 1125-1140 (2018).
5. H. Sha *et al.*, The ER-associated degradation adaptor protein Sel1L regulates LPL secretion and lipid metabolism. *Cell Metab* **20**, 458-470 (2014).
6. S. Sun *et al.*, Sel1L is indispensable for mammalian endoplasmic reticulum-associated degradation, endoplasmic reticulum homeostasis, and survival. *Proc Natl Acad Sci U S A* **111**, E582-591 (2014).
7. Z. Zhou *et al.*, Endoplasmic reticulum-associated degradation regulates mitochondrial dynamics in brown adipocytes. *Science* **368**, 54-60 (2020).
8. H. Mao, G. H. Kim, L. Pan, L. Qi, Regulation of leptin signaling and diet-induced obesity by SEL1L-HRD1 ER-associated degradation in POMC expressing neurons. *Nature communications* **15**, 8435 (2024).
9. L. Qi, L. Yang, H. Chen, Detecting and quantitating physiological endoplasmic reticulum stress. *Meth Enzymol* **490**, 137-146 (2011).

Supplemental Figures and Figure Legends

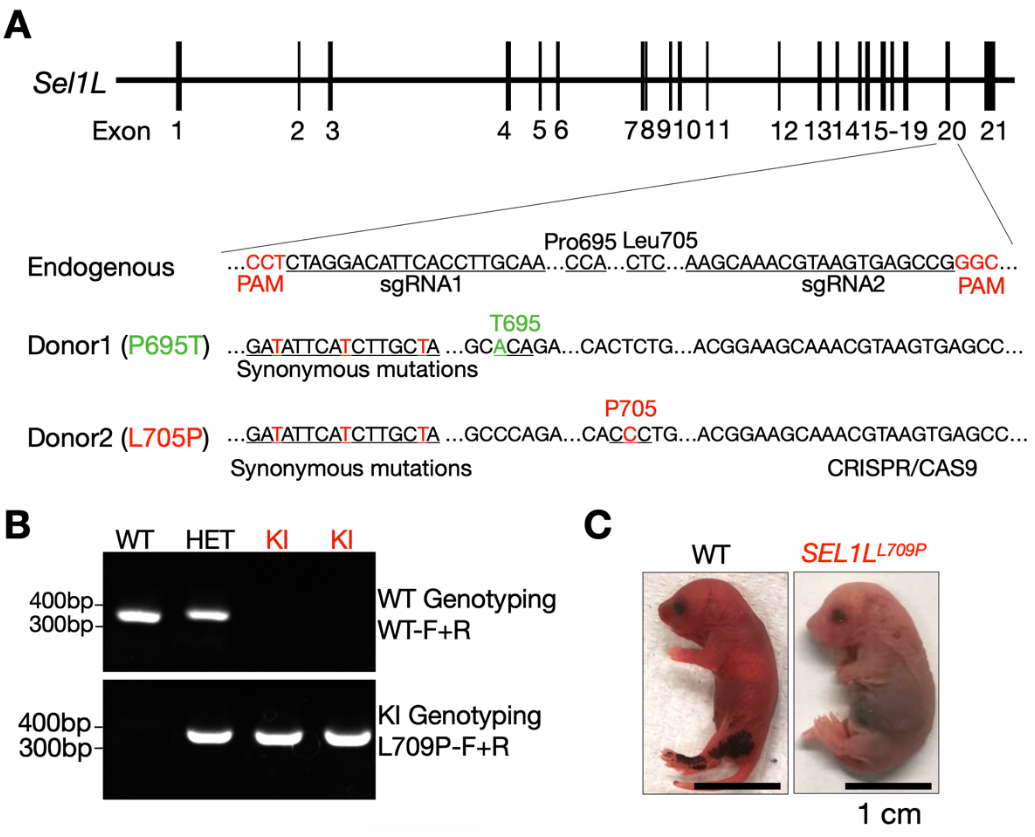

Figure S1. Generation of *Sel1L*^{L709P} and *Sel1L*^{P699T} KI mice.

(A) Schematic diagram showing CRISPR/Cas9-mediated gene editing. Mouse residues P695 and L705 correspond to human P699 and L709, respectively.

(B) Representative genotyping PCR showing the identification of WT, heterozygous (HET), and homozygous KI alleles. L709P and P699T KI mice shared the same primer pairs for genotyping.

(C) Gross examination of deceased *Sel1L*^{L709P} pups reveals no external malformations.

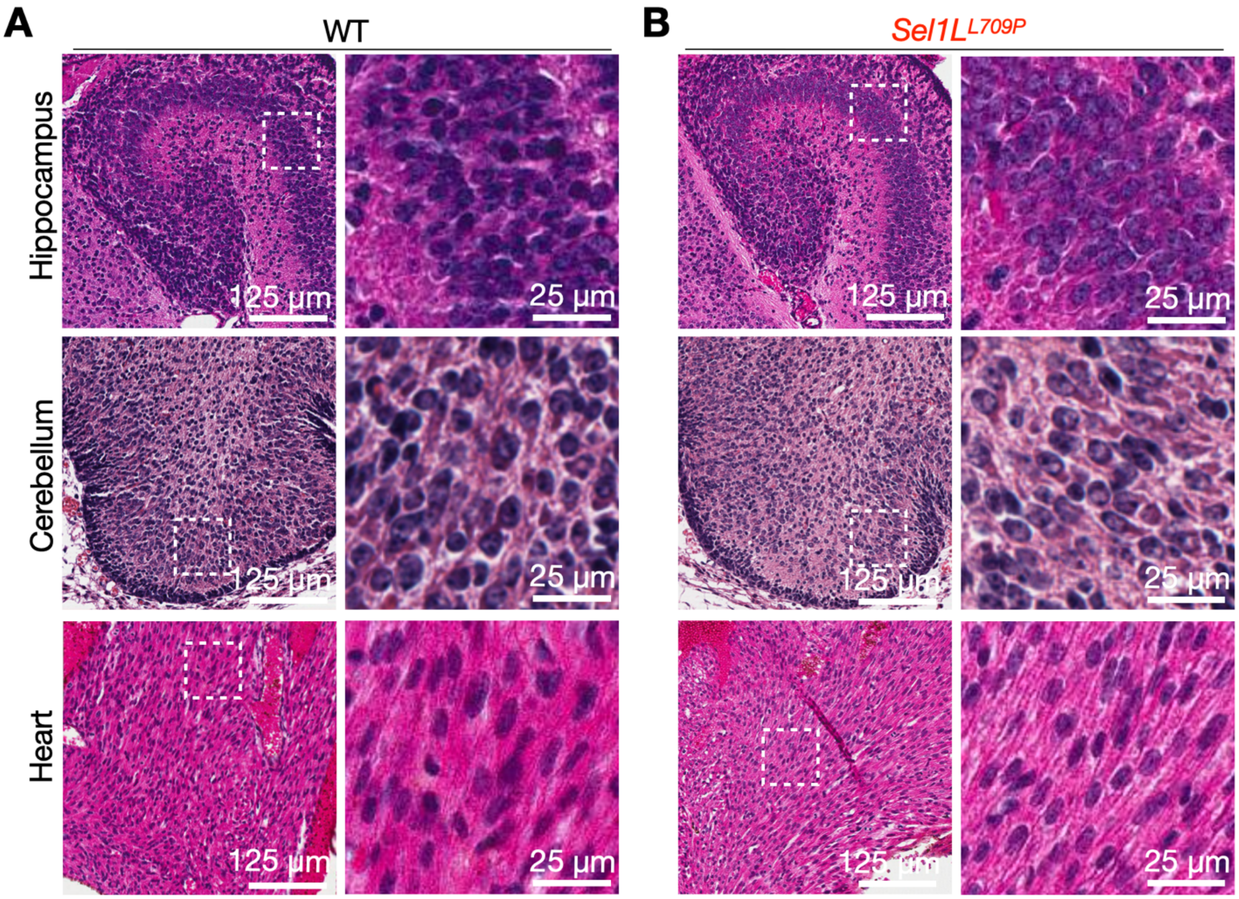

Figure S2. Histological examination of WT (A) and *Sel1L*^{L709P} KI (B) neonatal tissues including hippocampus, cerebellum and heart. Representative images from n = 3 P0 mice per genotype with no obvious abnormalities observed.

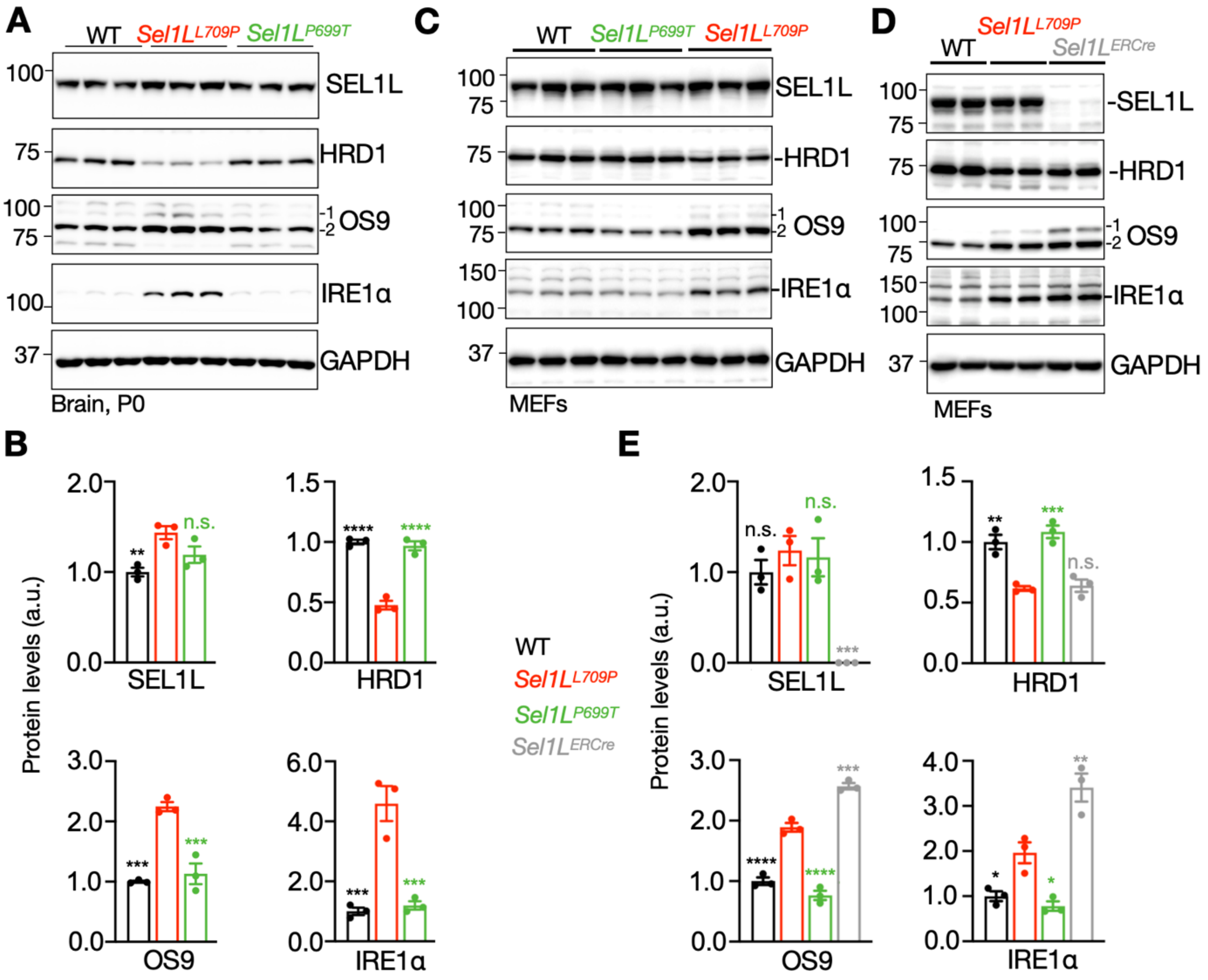

Figure S3. Impaired ERAD function in *Sel1L*^{L709P} KI MEFs and brains.

(A-B) Immunoblot analyses of ERAD-related proteins in whole-brain lysates from WT and two KI mice (A). Quantification is shown in (B), normalized to the loading control GAPDH. n = 3 mice per group.

(C-E) Immunoblot analyses of ERAD components and substrates in WT, *Sel1L*^{L709P} and *Sel1L*^{P699T} and *Sel1L*^{ERCre} MEFs (C-D). Quantification is shown in (E), normalized to GAPDH. n = 3 mice per group.

Values are shown as mean ± SEM. Statistical comparisons are made relative to *SEL1L*^{L709P}**.** n.s., not significant; *p < 0.05; **p < 0.01; ***p < 0.001; ****p < 0.0001 using one-way ANOVA with Dunnett's multiple comparisons test.

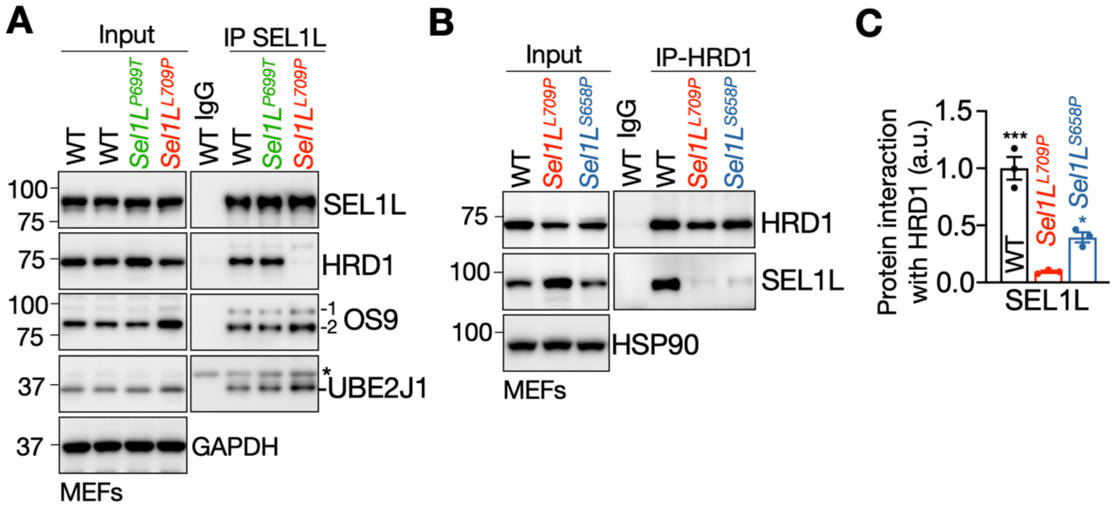

Figure S4. SEL1L L709P mutation abolishes SEL1L-HRD1 interaction *in vitro*.
(A) Co-IP of endogenous SEL1L from MEFs of WT, *Sel1L*^{P699T}, and *Sel1L*^{L709P} mice, followed by immunoblotting for ERAD components. Quantification is shown in Fig. 5E, normalized to SEL1L. Non-specific bands are indicated by asterisks.
(B-C) Co-IP of endogenous HRD1 from MEFs of the indicated genotypes, analyzed for interaction with SEL1L (B). Quantification is shown in (C), normalized to HRD1 levels; n = 3 mice per group. HSP90, a loading control. Asterisk indicates a non-specific band.
Values are shown as mean ± SEM. Statistical comparisons are made relative to *SEL1L*^{L709P}. n.s., not significant; *p < 0.05 and ***p < 0.001 using one-way ANOVA with Dunnett's multiple comparisons test.

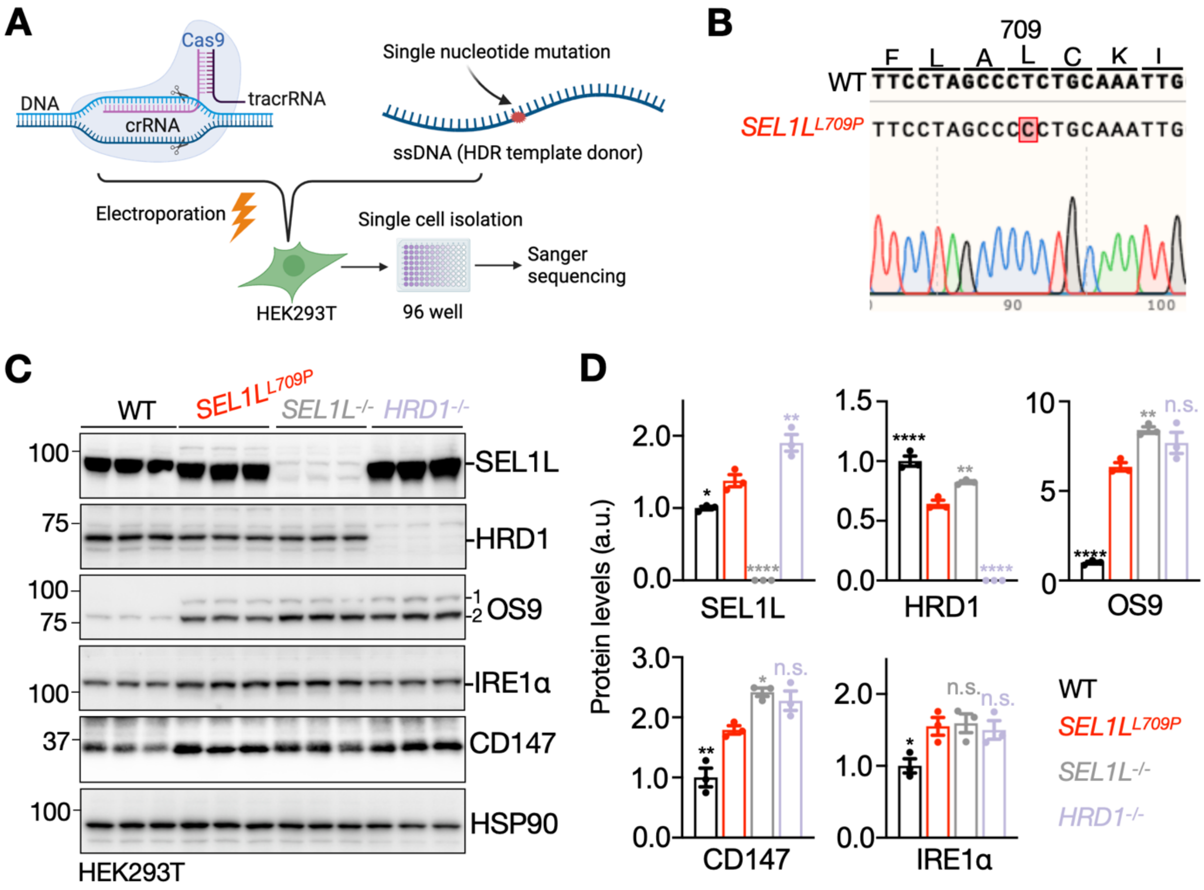

Figure S5. SEL1L L709P mutation abolishes SEL1L-HRD1 interaction in human cells.
(A) Schematic of CRISPR/Cas9-mediated KI of the L709P mutation in HEK293T cells.
(B) Sanger sequencing confirmation of biallelic KI cell lines.
(C-D) Immunoblot analyses of ERAD-related components and substrates in WT, *SEL1L*^{L709P}, *SEL1L*$^{-/-}$, and *HRD1*$^{-/-}$ HEK293T cells (C). Quantification is shown in (D), normalized to the loading control HSP90; n = 3 independent samples.
Values, mean ± SEM. Statistical comparisons are made relative to *SEL1L*^{L709P}**.** n.s., not significant; *p < 0.05; **p < 0.01; ****p < 0.0001 using one-way ANOVA with Dunnett's multiple comparisons test.

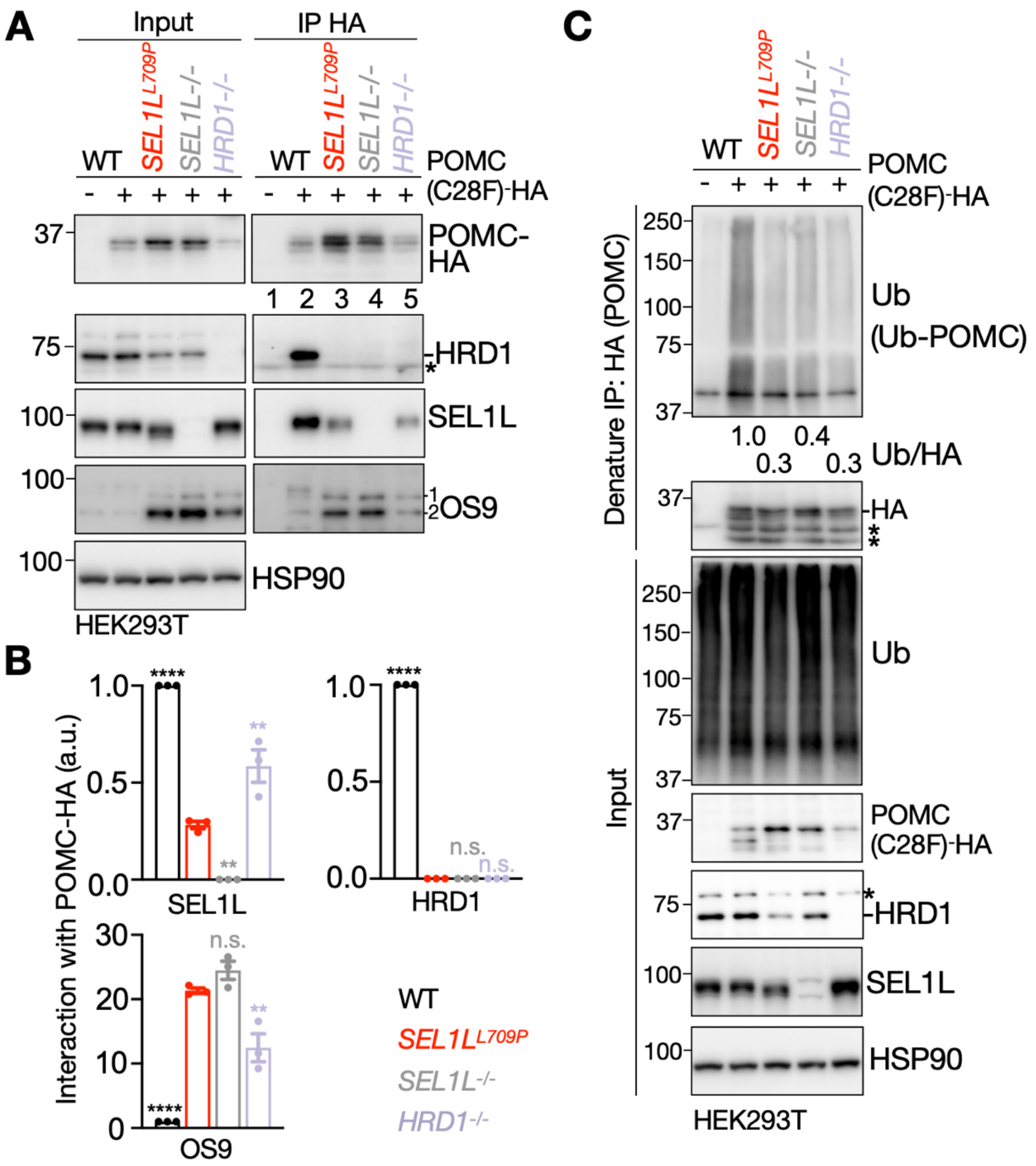

Figure S6. The SEL1L-HRD1 interaction is essential for substrate recruitment and ubiquitination. (A-B) Co-immunoprecipitation of POMC (C28F)-HA in transfected *SEL1L*$^{-/-}$ and *SEL1L*^{L709P} KI HEK293T cells. HSP90, a loading control. Asterisk indicates a non-specific band. Quantification is shown in (B). n = 3 independent samples.

(C) Immunoblot of ubiquitinated POMC (C28F)-HA showing impaired substrate ubiquitination in *HRD1*$^{-/-}$, *SEL1L*$^{-/-}$ and *SEL1L*^{L709P} KI HEK293T cells treated with MG132 for 4 hrs. n = 3 independent samples. Asterisks, non-specific bands.

Values are mean ± SEM. Statistical comparisons are made relative to *SEL1L*^{L709P}. n.s., not significant; **p < 0.01; ****p < 0.0001 using one-way ANOVA with Dunnett's multiple comparisons test.

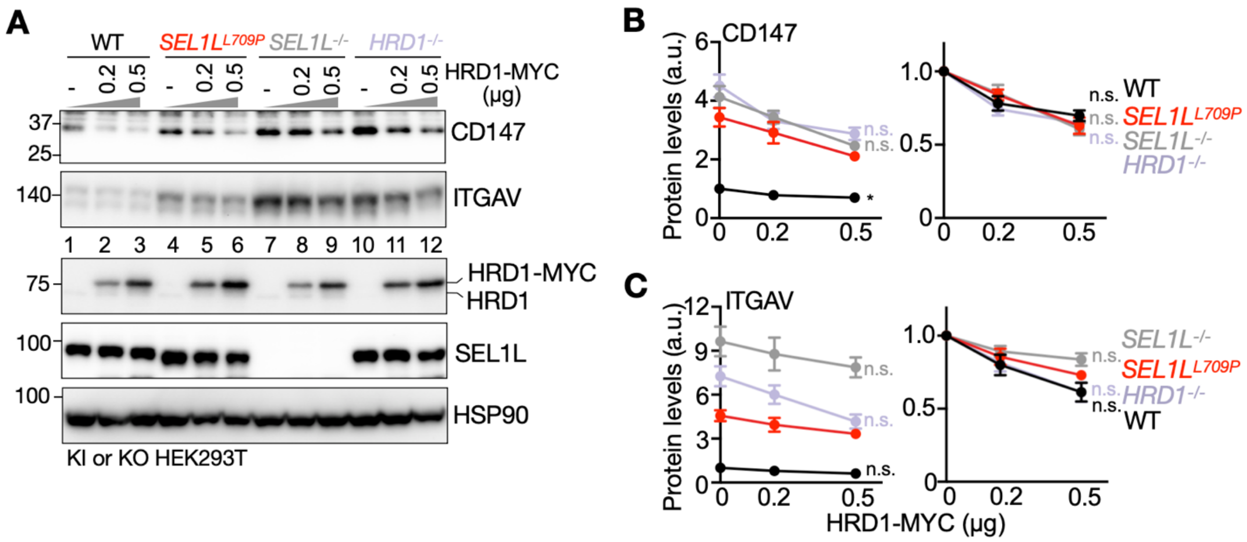

Figure S7. HRD1 overexpression reduces substrate accumulation in ERAD-deficient cells. (A-C) Western blot analyses of endogenous CD147 and ITGAV in KO or KI HEK293T cells transfected with increasing amounts of WT HRD1-MYC (A). Quantification is shown in (B) for CD147 and (C) for ITGAV. In B-C, the left graphs are normalized to each sample's own HSP90, while the right graphs include an additional normalization to the 0 µg/ml HRD1-MYC condition of the same genotype. n = 6-7 independent samples. Values are presented as mean ± SEM. Statistical comparisons are made relative to substrate decay dynamics in HRD1-transfected *SEL1L*^{L709P} cells. n.s., not significant; *p < 0.05 determined by simple linear regression.
